# Orthogonal proteomics methods warrant the development of Duchenne muscular dystrophy biomarkers

**DOI:** 10.1186/s12014-023-09412-1

**Published:** 2023-06-12

**Authors:** Camilla Johansson, Helian Hunt, Mirko Signorelli, Fredrik Edfors, Andreas Hober, Anne-Sophie Svensson, Hanna Tegel, Björn Forstström, Annemieke Aartsma-Rus, Erik Niks, Pietro Spitali, Mathias Uhlén, Cristina Al-Khalili Szigyarto

**Affiliations:** 1 Department of Protein Science, School of Chemistry, Biotechnology and Health, KTH - Royal Institute of Technology, Stockholm, Sweden; 2 Science for Life Laboratory, KTH - Royal Institute of Technology, Solna, Sweden; 3 Mathematical Institute, Leiden University, Leiden, The Netherlands; 4 Department of Human Genetics, Leiden University Medical Center, Leiden, The Netherlands; 5 Department of Neurology, Leiden University Medical Center, Leiden, The Netherlands

**Keywords:** Duchenne muscular dystrophy, Serum biomarkers, Biomarker quantification, Sandwich immunoassay, Mass spectrometry, Parallel reaction monitoring.

## Abstract

**Background:**

Molecular components in blood, such as proteins, are used as biomarkers to detect or predict disease states, guide clinical interventions and aid in the development of therapies. While multiplexing proteomics methods promote discovery of such biomarkers, their translation to clinical use is difficult due to the lack of substantial evidence regarding their reliability as quantifiable indicators of disease state or outcome. To overcome this challenge, a novel orthogonal strategy was developed and used to assess the reliability of biomarkers and analytically corroborate already identified serum biomarkers for Duchenne muscular dystrophy (DMD). DMD is a monogenic incurable disease characterized by progressive muscle damage that currently lacks reliable and specific disease monitoring tools.

**Methods:**

Two technological platforms are used to detect and quantify the biomarkers in 72 longitudinally collected serum samples from DMD patients at 3 to 5 timepoints. Quantification of the biomarkers is achieved by detection of the same biomarker fragment either through interaction with validated antibodies in immuno-assays or through quantification of peptides by Parallel Reaction Monitoring Mass Spectrometry assay (PRM-MS).

**Results:**

Five, out of ten biomarkers previously identified by affinity-based proteomics methods, were confirmed to be associated with DMD using the mass spectrometry-based method. Two biomarkers, carbonic anhydrase III and lactate dehydrogenase B, were quantified with two independent methods, sandwich immunoassays and PRM-MS, with Pearson correlations of 0.92 and 0.946 respectively. The median concentrations of CA3 and LDHB in DMD patients was elevated in comparison to those in healthy individuals by 35- and 3-fold, respectively. Levels of CA3 vary between 10.26 and 0.36 ng/ml in DMD patients whereas those of LDHB vary between 15.1 and 0.8 ng/ml.

**Conclusions:**

These results demonstrate that orthogonal assays can be used to assess the analytical reliability of biomarker quantification assays, providing a means to facilitate the translation of biomarkers to clinical practice. This strategy also warrants the development of the most relevant biomarkers, markers that can be reliably quantified with different proteomics methods.

**Supplementary Information:**

The online version contains supplementary material available at 10.1186/s12014-023-09412-1.

## Background

Comprising thousands of different proteins at concentrations spanning over several orders of magnitude, serum and plasma are complex biological samples, considered to mimic the health status of the individual they originate from. Blood samples provide a low-invasive, readily accessible material for the analysis of molecular markers, to be used as diagnostic, prognostic, disease progression or molecular therapy outcome biomarkers [1, 2]. Although many protein biomarkers have been discovered using high-throughput proteomics technologies, only a few of them have been validated and used in clinical practice [3–5] after approval by regulatory authorities [4].

The limited translation of biomarkers to medical care is partially attributed to poor reproducibility of results [6, 7]. Factors influencing reproducibility relate to the performance of the analytical methods employed, the inherent biological variability of non-identical samples as well as the statistical power of the study [8]. Furthermore, the analytical methods employed in biomarker studies are also affected by factors such as noise, limit of detection and accuracy that introduces technology dependent variability. While immuno-based technologies using antibodies are sometimes prone to cross-react with other proteins apart from the intended target [9], mass spectrometry-based methods cope with factors such as high false discovery rate and ion suppression effects [10, 11]. Furthermore, discovery studies often rely on high-throughput methods that provide relative quantification of biomarkers in contrast to the clinical practice, which requires absolute quantification of biomarkers for each patient [12]. Discovered molecular biomarkers need to be confirmed for a defined context of use in samples collected from large cohorts by using sensitive assays for absolute quantitative measurements before clinical validation is considered.

The discovery of protein biomarker candidates in the context of Duchenne muscular dystrophy has been successful, but no protein biomarker candidate has yet been validated, approved by regulatory authorities and translated to clinical use [13]. As DMD is a fatal, incurable progressive neuromuscular disease, the development of novel biomarkers can benefit patients and aid in the development of novel therapies [14]. The disease initially causes delay of motor milestones and as the disease progresses, loss of motor function, with complete loss of ambulation at an age between 8 and 14 [15] followed by symptoms of diaphragm and myocardium deterioration during adolescence with consequent decreased life expectancy [16]. Currently, diagnostic and disease progression assessments for the clinical management of DMD include a wide range of physical tests and blood tests with limited selectivity and sensitivity [17, 18]. Physical tests, although proven to be reliable may be influenced by factors other than the disease progression, such as motivation and the ability to follow instructions [19, 20]. Blood tests are routinely used to measure elevated total serum creatine kinase (CK) [6, 7] activity in patients affected by DMD. Although used currently as an early indication of certain muscular dystrophies, the specificity of CK is limited since it is ubiquitously expressed, elevated in most genetic and acquired forms of muscular dystrophy and influenced by physical exercise and other disease conditions [21, 22]. Skeletal muscle composition in terms of measured fat fraction by quantitative magnetic resonance imaging is currently considered a promising exploratory biomarker [23]. Three-point Dixon Magnetic Resonance Imaging analysis over time can provide disease progression estimates in DMD for use in clinical trials [24, 25]. Quantitative muscle/fat estimates are independent of patients’ motivation [26, 27] but are not part of the routine and long-term clinical follow-up, due to the lack of standardized protocols for image acquisition and data analysis, and the cost and the duration of the scans [28]. The lack of accurate and sensitive tests to monitor the disease state not only complicates the clinical management of the disorder but also obstructs the development and approval of novel therapies. This has urged researchers to find novel, more informative biomarkers for DMD.

Proteomics studies using different methodologies reported elevated blood levels of muscle-specific proteins in DMD patients, as well as proteins involved in energy metabolism, fibrosis (fibronectin) and inflammation [29–35]. Among them, matrix metalloproteinase-9 has been thoroughly assessed in one of the largest studies comprising samples from 3 independent clinical trials testing the effect of drisapersen [34, 36]. Previously, we identified and confirmed protein biomarkers associated with disease severity and progression in a multicohort study comprising both serum and plasma [37, 38]. Carbonic anhydrase 3 (CA3) has been shown to be a severity marker whereas malate dehydrogenase 2 (MDH2) was identified to be a potential predictor of disease milestone, associated with the time to loss of ambulation [37]. In addition, MDH2, ankyrin repeat domain 2 and collagen alpha-1(I) chain (COL1A1) were associated with corticosteroid treatment. Among the numerous biomarkers identified, few have been validated and confirmed for a specific context of use. In this paper, we propose an orthogonal strategy for the analytical validation of ten previously discovered biomarker candidates [29, 37, 38]. The biomarkers are quantified in serum samples collected longitudinally from DMD patients using parallel reaction monitoring mass spectrometry (PRM-MS). In addition, cross-validation of the biomarker quantification assays with the PRM-MS assay is corroborated with sandwich immunoassays for two of the biomarkers. This orthogonal validation strategy simultaneously validates the accuracy of both methods and provides analytical validation for biomarker behaviors observed in the discovery trial.

## Methods

### Sample collection and pre-processing

Eighty-four serum samples from a longitudinal cohort consisting of 20 DMD patients and 12 healthy control patients (Table 1), along with patient data, were collected at Leiden University Medical Center (LUMC) using standardized sample collection and handling protocols. The samples together with information about diagnosis, ambulation state and age at the time of sample retrieval, were collected from recruited individuals only if informed consent was obtained and the study was conducted following the Declaration of Helsinki. Collection of the samples was approved by the LUMC Commissie Medische Ethiek and the study was approved by Regionala etikprövningsnämnden Stockholm, Sweden (ref. 2018/1859-31/1). Samples were aliquoted and divided between two laboratories for biomarker quantification and stored at -80 °C prior to the analysis. The total protein concentration of each sample was measured at 5 different dilutions using the Pierce™ BCA Protein assay (23,225, Thermo Scientific) according to the manufacturer’s instructions.

**Table 1:** Summary of patients and samples included in the longitudinal cohort

Biomarker quantification method	Number of DMD patients				Number of samples	
	Total	3 time-points	4 time-points	5 time-points	DMD patients	Controls
PRM-MS	20	11	6	3	72	12
Sandwich immuno-assay	20	12	5	3	71*	12

*One sample was not analyzed by sandwich immunoassay in contrast to PRM-MS due to limited availability

PRM-MS and sandwich immunoassay analysis were performed using serum from the LUMC cohort (best time point distribution). Included in the analysis are all controls and longitudinally collected samples at 5 time points, 4 time points, and 3 time points from DMD patients.

### Spectral library and PRM method generation

Protein epitope signature tags (PrESTs) produced as stable ^13^C^15^N-isotope labeled standards (SIS-PrESTs) were used for the analysis of serum proteins by mass spectrometry [39]. Only PrESTs that generate 3–5 unique peptides upon proteolytic cleavage with trypsin were considered. Briefly, SIS-PrESTs were expressed using an auxotrophic *Escherichia coli* strain in the presence of ^13^C and ^15^N isotopes labeled lysine and arginine, purified and quantified using the N-terminal quantification tag (QTag). Absolute quantification was performed with non-labeled ultra-purified QTag standard and calculated using the median ratio of three proteotypic peptides [39, 40].

100 fmol/SIS-PrESTs solubilized in solvent A (3% Acetonitrile (ACN), 97% H_2_O, 0.1% Formic acid (FA)) was loaded onto an Acclaim PepMap 100 trap column (75 μm × 2 cm, C18, 3 μm, 100 Å, Thermo Scientific) using an UltiMate 3000 binary RS nano-LC system with an EASY-Spray ion source, washed 5 min at 5 µl/min with 100% of solvent A, and then separated by a PepMap RSLC C18 column (75 μm x 50 cm, 2 μm, 100 Å, Thermo Scientific) at 35 °C. The peptides were eluted with a linear 90 min gradient of 3–35% solvent B (95% ACN, 5% H_2_O, 0.1% FA) at a flow rate of 0.300 µl/min followed by a 7 min increase to 99% solvent B. The column was washed for 11 min with 99% solvent B and then equilibrated for 5 min with 3% solvent B. Subsequent quantification the SIS-PrESTs were pooled in equimolar amounts and used for quantification of serum proteins [39].

Spectral library generation was performed using the SIS-PrEST pool with the Top5MS method according to Edfors et al. 2019 [39] with minor modifications. The MS1 scan with 60,000 resolution at 200 m/z (AGC target 3e^6^, range 300 to 1,600 m/z, injection time 100 ms) was followed by five MS/MS scans at 60,000 resolution at 200 m/z (AGC target 1e^6^, range 200 to 2,000 m/z, injection time 150 ms) with isolation window 2 m/z, normalised collision energy (NCE) 28 and dynamic exclusion of 15 s. Raw files were searched in MaxQuant version 1.5.2.8 (Cox & Mann, 2008), using the search engine Andromeda against SIS-PrEST sequences with an *E. coli* (BL21 UniprotID: #UP000002032) background in order to limit false-positive hits against SIS-PrEST peptides. Arg10 and Lys8 were chosen as heavy labels with a maximum of 3 labels. The enzyme specificity was set to trypsin and a maximum of 2 missed cleavages were allowed. Oxidation on methionine and N-terminal acetylation were set as variable and carbamidomethylation on cysteine was set as a fixed modification. The false discovery rate (FDR) that was determined by a reverse database was set to 0.01 for both peptide and protein levels, whereas the peptide minimum length was set to 7 amino acids. Identified peptides were further processed by only allowing proteotypic peptides mapping to one single human gene (defined by SwissProt) with precursor charges + 2 or  + 3, also excluding the last peptides of the SIS-PrEST sequence, peptides with more than 1 missed cleavage and with possible post-translational modifications. Peptides for biomarker quantification were selected by evaluating their endogenous signal in a pooled serum sample from three subjects. The final number of peptides was limited based on their retention time to allow for at least 10 points across the chromatographic peak (approx. 20 s). As the MS method cycle time in the final PRM method was 6.1 s the number of concurrent precursors was limited to 6.

### Protein quantification using parallel reaction monitoring

Two µl of serum samples was transferred into 50 mM AmBic on the CyBio® Selma liquid handler (Analytik Jena Ag, Germany) and mixed with 8 µl of SIS-PrEST pool that represented an approximately 1:1 (Light:Heavy, L:H) peptide ratio to the endogenous levels in serum. The proteins were denatured with 1% sodium deoxycholate (SDC), reduced with 10 mM DTT for 20 min at 56 °C, alkylated with 50 mM CAA and incubated in the dark for 20 min. Proteins were digested with LysC and trypsin for 1 h and overnight, respectively. The digestion was quenched by adding TFA to a final concentration of 0.5% (v/v) and SDC precipitated for 30 min at room temperature and then centrifuged for 10 min at 3,273 rcf on an Allegra X-12R centrifuge (Beckman-Coulter, Brea, CA, USA). The peptides were cleaned as described previously [41], vacuum-dried and stored at -20 °C. For LC/MS/MS a total of 1 µg peptides were first loaded onto the trap column, washed 5 min at 5 µl/min with 100% solvent A, and then separated by a PepMap RSLC C18 column (75 μm x 25 cm, 2 μm, 100 Å, Thermo Scientific). The peptides were eluted with a linear 33 min gradient of 3–30% solvent B at a flow rate of 0.400 µl/min followed by a 2 min increase to 43% and then a 1 min increase to 99% of solvent B. The column was washed with 99% solvent B for 8 min, at a flow rate of 0.650 µl/min and then equilibrated with 1% solvent B, for 10 min, at a flow rate of 0.400 µl/min. Seven replicates were performed to test method suitability and repeatability across the sample plate.

For sample analysis each full MS scan at 60,000 resolution (AGC target 3e^6^, mass range 350-1,600 m/z and injection time 110 ms) was followed by 20 MS/MS scans at 60,000 resolution (AGC target 2e^5^, NCE 27, isolation window 1.5 m/z and injection time 300 ms). The PRM method was defined by a scheduled (3 min windows) PRM isolation list that contained 34 paired light and heavy peptide precursors. The raw MS-files were processed in Skyline version 4.1. The ratio between endogenous and SIS-PrEST peptide was calculated for each peptide as the summed area intensity over the retention time. Peak integration was performed automatically, and peak boundaries were adjusted in cases where the peak apex had not been integrated by Skyline. At most, the 10 most intensive fragments (in the m/z range of 50 to 1,500) from the spectral library per precursor were used for quantification. All results with ratio dot product (rdotp) values less than 0.6 were excluded. One peptide with a very low endogenous signal and high variability in replicates was removed. The absolute peptide concentrations were calculated by using the absolute and known amounts of spiked in protein standards, and the median values of peptides per protein standard were calculated for the absolute protein concentrations or in the case of only a single peptide per protein, the peptide value was used. Serum protein concentration measurements were probabilistic quotient normalized over all targets (including seven non-disease related control proteins) divided by the mean total serum protein concentration for the cohort to account for sample variation introduced by storage conditions and accumulated pipetting errors. Normalized concentrations were subsequently log2-transformed and analyzed using a linear mixed effects model with random intercept [42] where patient group, age and interactions between them were included as covariates. Hypothesis testing was performed using an F test with degrees of freedom determined using Satterwhite’s correction [43]. Multiple testing corrections were implemented using the Benjamini-Hochberg method [44].

### Construction of anti-CA3 sandwich immunoassay

A sandwich immunoassay for CA3 was built using Luminex xMAP technology (Luminex Corp.). A polyclonal anti-CA3 antibody produced in-house [45] was used as capture antibody, whereas a polyclonal anti-CA3 antibody (15197-1-AP, Proteintech) was used as detection antibody. Both antibodies are validated by Western Blot and Immunohistochemical staining. To construct the CA3 capturing module, MagPlex beads were coupled with anti-CA3 antibody as described previously [38]. As negative controls, 100 µl of MES (0.1M 2-(N-morpholino) ethanesulfonic acid, pH 4.5) buffer was added to a second activated bead and 17.5 µg/ml bovine serum albumin was added to a third bead. The activated beads were incubated with antibody/control solutions for 2 h at 800 rpm, washed in PBS-0.05% Tween 20 (PBST) and blocked in 50 µl 5% w/v bovine serum albumin in PBST. Anti-CA3 antibody beads and negative control beads were pooled at equivalent volumes (anti-CA3 bead pool).

In parallel, the detection antibody was biotinylated on solid-phase using Protein A coated magnetic beads (Dynabeads, Invitrogen) and EZ-link NHS-PEG4-biotin (ThermoFisher). In short, 2 µg of antibody was captured on 300 µg of beads according to manufacturer’s instructions, and incubated with 60 µl of 86 µg/ml EZ-link NHS-PEG4-biotin in PBS with 14% DMSO for 2 h at 4 °C. The biotinylated antibodies were eluted in 20 µl 0.2 M acetate, pH 3.2, and neutralized to pH 5.5 using 1 M Tris-HCl and PBST.

### Protein quantification using anti-CA3 and anti-LDHB sandwich immunoassays

Anti-CA3 sandwich immunoassay was performed with beads prepared as described above. Anti-LDHB sandwich immunoassay was performed using the LDH-B Human ProcartaPlex™ Simplex Kit (EPX010-12262-901, Invitrogen) according to the manufacturer’s instructions, but with modifications. For the anti-CA3 sandwich assay, samples were diluted in assay buffer containing 0.5 mg/ml rabbit IgG and 1:1000 ProClin™300 in PBST in 6 replicates at different total serum protein concentrations spanning from 1.12 ± 0.12 mg/ml to 0.11 ± 0.01 mg/ml. Recombinant human CA3 (ab173078, Abcam) was diluted to 12 different concentrations between 1.1 and 96 ng/ml in assay buffer supplemented with the corresponding amount of guinea-pig serum. For the anti-LDHB sandwich immunoassay, samples were diluted in assay buffer containing 0.5 mg/ml rabbit IgG 0.025% (w/v) polyvinylalcohol 0.04% (w/v) polyvinylpyrrolidone (Sigma) 0.005% (w/v) casein (Sigma) 1:1000 ProClin™300 PBS-0.05% (vv) Tween 20 to a total serum protein concentration of 0.84 ± 0.09 mg/ml. Recombinant LDHB was diluted in assay buffer according to manufacturer’s instructions. 50 µl of diluted samples and standards were heat-treated at 56 °C for 30 min, mixed with beads (anti-CA3 or LDH-B Human ProcartaPlex™ Simplex beads), and incubated overnight at 4 °C and 800 rpm. Following incubation, the beads were washed three times in PBST using a plate magnet and incubated with 7.5 ng biotinylated detection antibody in 25 µl PBST for 1 h at room temperature and 800 rpm. The sandwich assay was washed three times in PBST and visualized by incubating the assay with 1.3 µg/ml streptavidin R-phycoerythrin for 20 min prior to analysis. The samples were analysed in a Luminex® 100/200™ System flow cytometer with xPONENT software (Luminex Corp.) and median fluorescence intensities (MFI) obtained for each sample and protein standard. Five parametric log-logistic regression [46] was used to model the standard curves. For each plate, a linear region for concentration measurements was defined to span between two standard deviations above the mean background MFI and 90% of the MFI of the highest measured standard sample. The concentration of the CA3 protein of 29.6 kDa or LDHB protein of 37 kDa kDa (www.proteinatlas.org) was normalized to total serum protein concentrations. All plots and statistical calculations were performed in R software [47].

## Results

### Study rationale

**Fig. 1:**
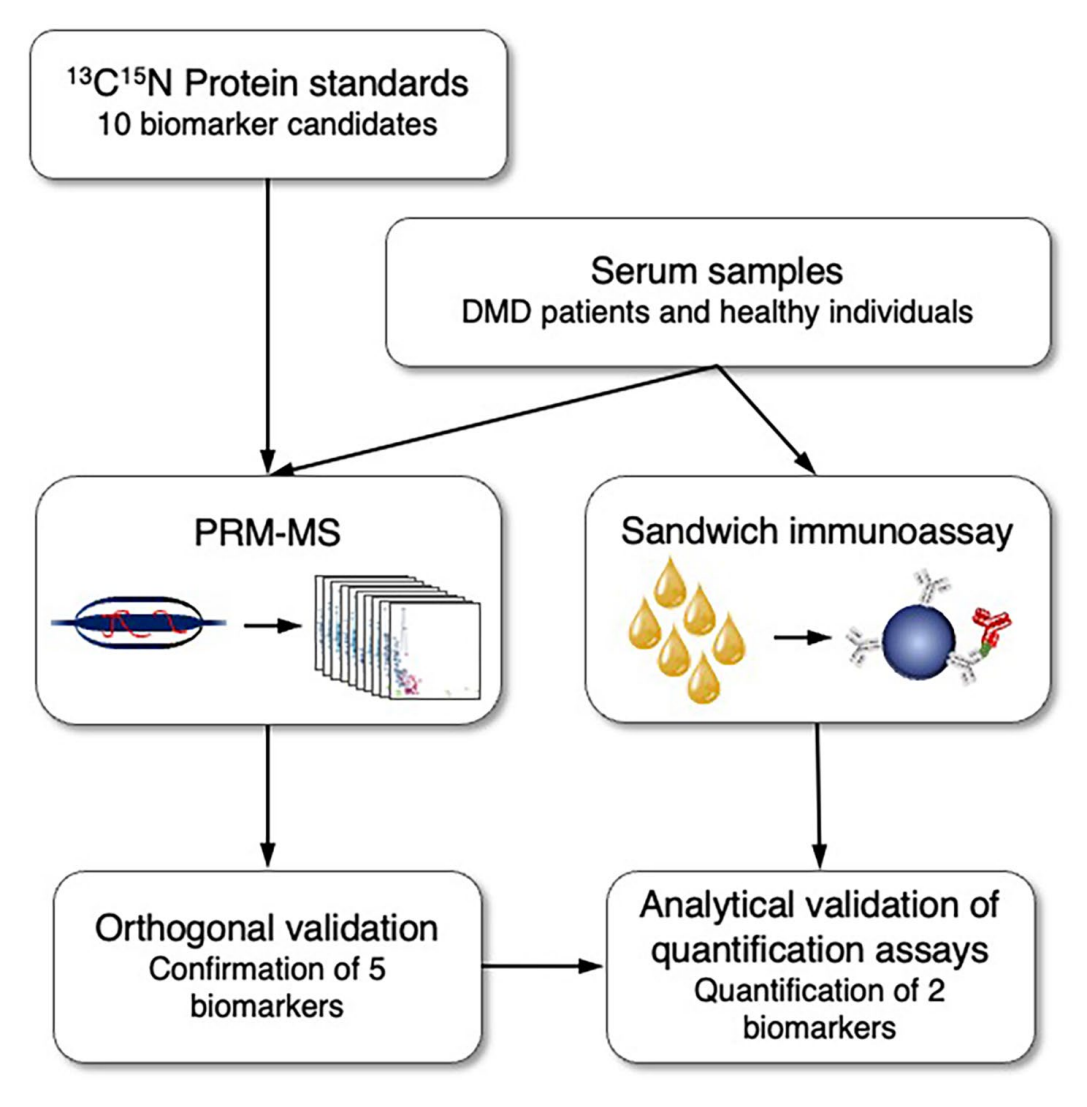
Schematic overview of the analysis

Although several discovered protein biomarkers have been confirmed to be associated with DMD, none of them have been successfully validated for translation to clinical practice [48]. The difficulty in translating biomarkers from discovery to clinical use is often associated with the lack of biological and analytical validation [49, 50]. In this study, orthogonal analytical validation was performed to confirm the association of previously discovered biomarker candidates with DMD and loss of ambulation. Ten biomarker candidates that correlated with disease progression, severity or that were predictive of likelihood of future disease milestones were selected for this study. The biomarkers CA3 [29, 34, 37, 38], MDH2 [29, 37, 38], COL1A1 [29, 37, 38], myosin light chain 3 (MYL3) [29, 37, 38], troponin type T3 (TNNT3) [29, 37, 38], electron transfer flavo-protein A (ETFA) [29, 37, 38], lactate dehydrogenase B (LDHB) [37, 38], nestin (NES) [37, 38], microtubule-associated protein 4 (MAP4)[37, 38] and fibrinogen gamma chain (FGG) [51], discovered by affinity-based proteomics methods, were analyzed using a mass spectrometry based method. For each biomarker candidate, protein fragments suitable to generate peptide standards for mass spectrometry were selected [52, 53] and produced as ^13^C^15^N-labeled SIS-PrESTs (Fig. 1). The SIS-PrESTs were subsequently spiked into samples and used for quantification of the biomarkers using PRM-MS. To achieve true orthogonal validation the PRM-MS were designed to detect the same fragment of the target as in the previous studies [29, 37, 38], The technical validity of the PRM-MS quantification assay was subsequently corroborated using a sandwich assay towards two of the targets, CA3 and LDHB.

In this study we used serum samples collected longitudinally from 20 DMD patients and cross-sectionally from 12 healthy patients (Table 1). The patient samples were collected at 3, 4 and 5 time points at approximately one-year intervals together with information about age at the time the sample was collected, ambulation status and treatment status.

### Orthogonal validation of biomarkers

Peptides corresponding to five out of ten potential DMD biomarkers were successfully detected and quantified. These findings orthogonally validate previously obtained results using affinity-based proteomics methods for MYL3 [29, 37, 38], CA3 [29, 34, 37, 38], COL1A1 [29, 37, 38], LDHB [37, 38], FGG [51]. Quantification of CA3, MYL3, and COL1A1 was based on 2 peptides whereas that of LDHB and FGG on one peptide. Quantification of biomarker candidates was achieved in 69 patient and 9 control samples. Biomarker detection was not achieved for three healthy individuals and one DMD patient most likely due to deterioration of sample components or low target concentration. In addition, one patient sample had FGG concentrations comparable to those of plasma samples and was excluded from subsequent analysis. Peptides corresponding to MDH2, ETFA, NES and MAP4 were not detected in any samples and TNNT3 was detected in only a small subset of samples and was therefore excluded from further analysis. For the biomarker candidates detected by mass spectrometry, serum concentrations were calculated by comparison of signal intensities of detected peptides originating from the sample with those originating from the spiked-in SIS-PrESTs. At least 3 unique peptides were used for quantification of the biomarker candidates (Table 2).

**Table 2:** Serum biomarker concentrations in healthy individuals and DMD patients

	DMD patients					Healthy controls				
Biomarker candidate	Min. value	25% quartile	Median value	75% quartile	Max. value	Min. value	25% quartile	Median value	75% quartile	Max. value
**MYL3 (ng/ml)**	5.7	50.1	117.9	234.7	708.9	3.6	6.7	12.4	21.7	45.3
**CA3 (ng/ml)**	360	1,091	2,272	3,189	10,257	16.5	31.1	63.9	91.6	397.6
**FGG (mg/ml)**	2.2	4.7	10.7	20.0	85.2	0.5	1.7	3.2	3.9	5.7
**LDHB (mg/ml)**	0.8	1.6	2.7	5.0	15.1	0.4	0.7	0.9	1.0	1.4
**COL1A1 (ng/ml)**	36	175	418	824	2,312	24	100	343	824	1,767

﻿Concentration of biomarker candidates in serum estimated by PRM-MS in healthy individuals and DMD patients.

Median concentrations of CA3 and MYL3 had the highest fold change between patients and controls, 35- and 9- fold respectively. COL1A1 had the lowest detected fold change of 1.2. Serum levels of CA3 and MYL3 ranged between 360 and 10, 257 ng/ml and 5.7 and 708.9 ng/ml in DMD patients and between 16.5 and 397.6 ng/ml and 3.6 ng/ml and 45.3 ng/ml in healthy individuals. LDHB varied between 0.8 and 15.1 ng/ml in DMD patients and between 0.4 and 1.34 mg/ml in controls. In contrast, COL1A1 ranged between 36 and 2,312 ng/ml in DMD patients and 24 and 1,757 ng/ml in serum from control patients. The serum concentration of FGG had a narrower variation interval and varied between 2.2 and 85.2 µg/ml in DMD patients and 0.5 and 5.7 µg/ml in healthy individuals.

To confirm that the biomarker variation was not due to sample quality the total protein concentration in each serum sample was measured. The total serum protein concentration varied between 25 and 127 g/L (Supplementary Fig. 1A) whereas the median total protein concentration for the cohort was measured to 75 g/L. The total protein concentration in serum did not correlate with age or with the disease pathology when comparing DMD patients with control samples from donors in the same age range as the patients (Supplementary Fig. 1B). However, only 48.8% of the samples were within the reference span for healthy serum, 60–80 g/L [54], while 39.7% were above and 11.5% below the reference span. This unusually high variation in total serum protein concentration could be a consequence of prolonged storage and technical handling and it was hypothesized that this may have significantly increased the variation of target proteins within the cohort. To account for a potential bias in variation, all immunoassay analyses (CA3 and LDHB) were normalized to the total serum protein concentration and displayed as fmol target per µg total serum proteins. Target concentrations from PRM-MS were normalized using probabilistic quotient normalization (PQN) [55, 56], which accounts for global accumulated technical variation in serum protein concentration up until the time point of data acquisition by re-scaling all target concentrations by a sample-specific scaling factor. Post PQN, concentrations were divided by the cohort mean total serum protein concentration, 75 g/L, to obtain a unit comparable to that from CA3 and LDHB sandwich immunoassays. These normalized values were used as biomarker concentrations in subsequent analysis [57, 58]. Storage and patient-related variation could be assumed to be the same in both PRM-MS analysis and sandwich immunoassays, as samples were thawed and aliquoted for both technologies simultaneously. Hence, differences in measured biomarker concentrations between the two assay types are assumed to reflect technological differences and variation introduced during sample preparation.

**Fig. 2:**
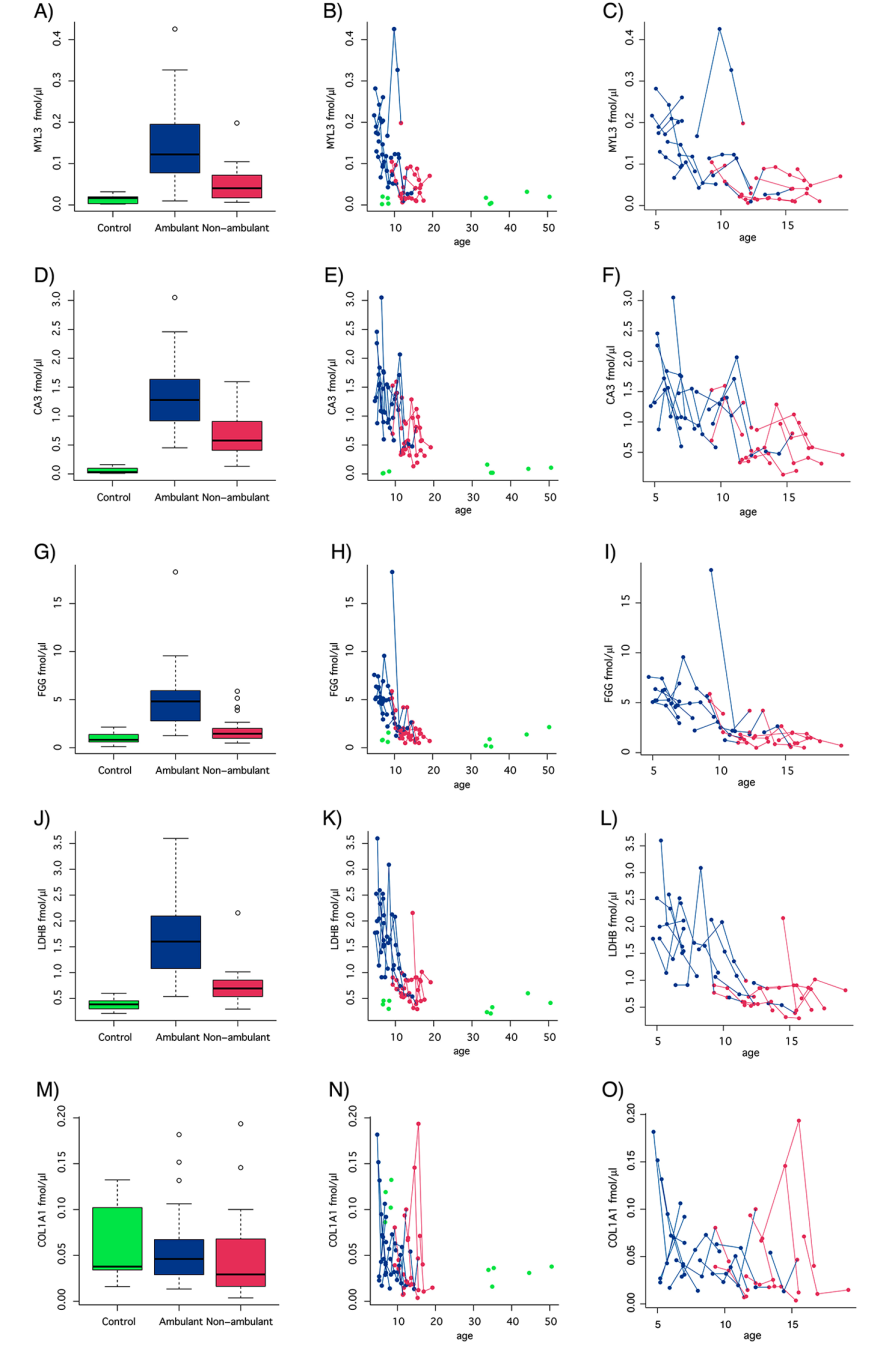
Serum concentrations of biomarker candidates in DMD patients and healthy individuals. Protein concentrations measured using PRM-MS were plotted with respect to ambulation status and age. Boxplots represent comparisons of MYL3 (A), CA3 (D), FGG (G), LDHB (J) and COL1A1 (M) concentrations in samples from 37 ambulant patients (blue), 32 non-ambulant patients (red), and 9 healthy individuals (green). Spaghetti plots represent MYL3 (B and C), CA3 (E and F), FGG (H and I), LDHB (K and L) and COL1A1 (N and O) concentration trajectories with respect to age with (C, E, H, K and N) and without (C, F, I, L and O) biomarker concentrations measured in healthy individuals. The concentrations are calculated as fmol target protein per µg of total serum proteins, and normalized against levels of non-disease related proteins to account for pipetting errors and water content variation across samples

Serum concentrations of MYL3, CA3, FGG and LDHB were elevated in DMD patients compared to age-matched healthy individuals (Fig. 2 plot A, D, G, J and M) and followed decreasing trajectories with increasing age in the patient cohort (Fig. 2 plot C, F, I, L and O) in comparison to the controls (Fig. 2 plot B, E, H, K, N), confirming previous experimental results (Supplementary Fig. 2) and conclusions [29, 37, 38]. The Spearman’s correlations for MYL3, CA3 and LDHB were 0.92, 0.9 and 0.83, respectively. In contrast, no correlation between the two measurements was observed for COL1A1. Serum levels of COL1A1, as shown in the affinity-based studies also had less distinct separation of patient groups and healthy controls (Fig. 2 plot I, J). The association of MDH2 [29, 37, 38], TNNT3 [29, 38], ETFA [29, 38], NES [38] and MAP4 [38] with DMD, as reported previously, was not confirmed by mass spectrometry.

**Table 3:** Evaluation of the protein concentration association with muscle function

		Adjusted P-values				
	Comparisons	MYL3	CA3	FGG	LDHB	COL1A1
	DMD patients vs. controls	**3.48E-06**	**7.86E-15**	**7.32E-08**	**1.21E-11**	1.63E-01
No age	Ambulant DMD patients vs. controls	**1.67E-06**	**7.34E-14**	**3.03E-07**	**5.88E-11**	5.62E-01
effect	Non-ambulant DMD patients vs. controls	**9.60E-05**	**4.80E-12**	**2.96E-03**	**2.43E-04**	1.06E-01
	Ambulant versus non-ambulant vs. patients	**4.92E-02**	**1.01E-02**	**2.17E-05**	**1.57E-07**	1.21E-01
	DMD patients vs. controls	**1.07E-06**	**5.89E-16**	**4.93E-07**	**4.54E-10**	**4.29E-02**
Age	Ambulant DMD patients vs. controls	**2.12E-07**	**4.06E-16**	**2.49E-06**	**9.79E-10**	**4.88E-02**
effect	Non-ambulant DMD patients vs. controls	**1.67E-04**	**1.14E-14**	**9.16E-04**	**2.15E-03**	**1.96E-02**
	Ambulant versus non-ambulant vs. patients	7.07E-01	9.00E-01	8.22E-01	**2.92E-02**	9.00E-01

Adjusted *P*-values were obtained from the linear mixed model analysis with and without age as a confounding factor. Adjusted *P*-values for the comparisons between the different DMD patient groups, ambulant and non-ambulant, and healthy aged-matched individuals are displayed. Significant differences (defined as adjusted *P*-values < 0.05) are marked in bold.

To evaluate the association of serum protein concentrations with muscle function in DMD, a linear mixed effects model analysis, with random intercept and disease group while keeping ambulant and non-ambulant as fixed effects, was performed. The analysis showed that serum concentrations of all the quantified proteins were significantly elevated in DMD patients, both ambulant and non-ambulant patient groups, compared to the controls, except for COL1A1. The adjusted *P*-values for MYL3, CA3, FGG and LDHB were below 0.05 (Table 3). As age is a confounding factor, a linear mixed effects model analysis was also performed including age and interaction between age and disease group as fixed effects. All biomarkers significantly separated DMD patients, both ambulant and non-ambulant, from controls (adjusted *P*-values < 0.05). However, only LDHB was able to discriminate between ambulant and non-ambulant patients when age was controlled for, indicating that the difference between ambulant and non-ambulant patients could be explained by the age difference. Previous observations suggested that the serum abundance of biomarkers decreases as the disease progresses and with increasing age [37, 38]. To further explore the variation of biomarkers over time, linear mixed effects model analysis was used to estimate the biomarker variation slopes in ambulant and non-ambulant patients. All biomarkers decreased over time and had negative slopes in both the ambulant and non-ambulant patient groups (Table 4). The adjusted *P*-values of the slopes for ambulant patients were lower than 0.05 for all biomarkers except COL1A1. In contrast, only FGG had a significant slope (adjusted *P* = 0.0034) in the non-ambulant cohort. The difference between the slopes before and after loss of ambulation was not significant for any of the biomarker candidates analyzed (Table 4).

**Table 4:** Comparison of serum biomarker decreases with age.

	Slope		Adjusted *P*-values		
Biomarker candidate	Ambulant	Non-ambulant	Slope ambulant patients	Slope non-ambulant patients	Slopes difference
**CA3**	-0.1834	-0.1235	**0.0040**	0.1066	0.7456
**COL1A1**	-0.1506	-0.1105	0.0664	0.2516	0.7456
**FGG**	-0.1883	-0.2297	**0.0048**	**0.0034**	0.7456
**LDHB**	-0.1855	-0.0507	**0.0001**	0.2516	0.0909
**MYL3**	-0.2886	-0.1145	**0.0021**	0.2516	0.2834

Age slopes were estimated for ambulant and non-ambulant patient groups using a linear mixed effects model. Adjusted *P*-values were calculated to assess whether the biomarker concentration decline before and after loss of ambulation was significant. Significant separations (defined as adjusted *P*-values < 0.05) are marked in bold.

**Fig. 3:**
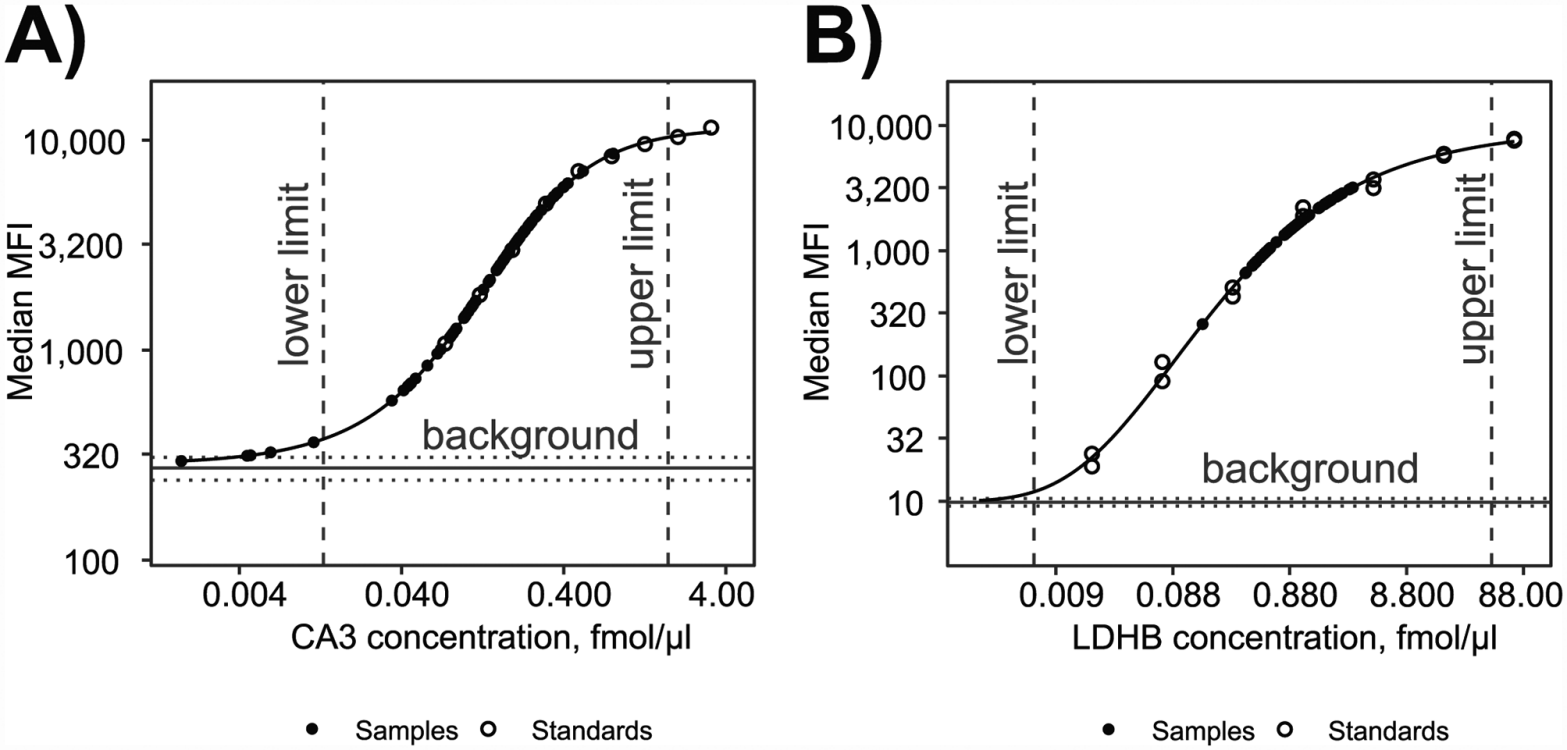
Quantification of CA3 (A) and LDHB (B) using immuno-based sandwich bead arrays. Measurements of protein standards are represented by open circles whereas those of patient samples with closed circles. Only samples with MFI values falling between the upper and lower limits were considered quantifiable. The lower limit was defined as samples with MFI values at least two standard deviations above the mean background MFI. The upper limit was defined as samples with MFI values of 90% or lower of the MFI value of the most concentrated recombinant protein standard sample. The gray horizontal lines denote the mean MFI value of all blank samples, with dotted lines indicating one standard deviation above and below the mean background MFI. Standard curves were estimated using five parametric log-logistic regression (5PL)

### Validation of the biomarker quantification

**Fig. 4:**
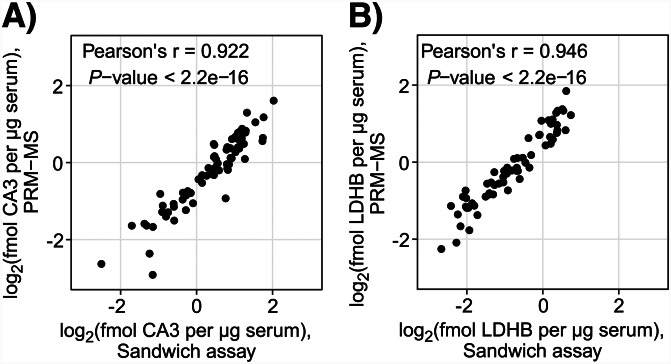
Comparison between CA3 (A) and LDHB (B) concentrations measured independently by PRM-MS and by sandwich suspension bead array. Each dot corresponds to a sample. Pearson’s correlation was used to determine the degree of similarity between the two methods with regard to the estimated CA3 serum concentration

Quantification of proteins using the PRM-MS method, can be influenced by several factors, such as the pretreatment of the sample, the efficiency of proteolytic digestion and generation of peptides, suboptimal analytical recovery of peptides during the analysis and ambiguity regarding the origin of redundant peptide sequences. To technically validate the quantification results, antibody-based quantification assays were employed for the 2 biomarker candidates, LDHB and CA3. Quantification of LDHB was performed using a commercially available sandwich immunoassay whereas quantification of CA3 was performed using an in-house developed assay. The CA3 quantification assay was developed using two anti-CA3 antibodies recognizing different protein epitopes as capture and detection reagents and recombinant CA3 in guinea pig serum as protein standards. Figure 3 shows standard curves generated with known concentrations of protein standards (open circles) and measured LDHB and CA3 concentrations in 71 serum (closed circles) samples (one sample omitted due to being limited in volume). CA3 standard curves for all sample replicates can be found in (Supplementary Fig. 3). Serum LDHB and CA3 measured using the affinity-based assay had similar concentrations as when quantified using the PRM-MS method (Fig. 4). Comparison of serum protein levels quantified using the sandwich immunoassay and the PRM-MS assay showed a Pearson correlation of 0.946 (*P*-value < 2.2e-16) for LDHB and 0.922 (*P*-value < 2.2e-16) for CA3. The results had a clear, high linear correlation between the two quantification methods for both biomarker candidates analyzed.

## Discussion

Attempts to compare results from different laboratories on biomarker discovery, indicate that biomarker candidates able to discriminate between DMD patients and healthy individuals are not all reproduced even when the same analytical assay and technology are used [34, 59]. The discrepancy can be explained by errors related to poor clinical accuracy (variability related to biological factors) and/or poor analytical accuracy (variability related to the detection assay). In previous studies we confirmed the association of biomarkers with DMD in samples collected at four geographically dispersed clinical hospitals. To minimize the discovery of false positive biomarkers due to biological variability, inter- and intracohort analysis were employed. In this paper, sensitive and specific PRM-MS is used for biomarker confirmation to circumvent errors/variability introduced by antibody-based proteomic methods used for the discovery of biomarkers [37, 38]. Longitudinally collected serum samples, previously analyzed as part of a larger longitudinal biomarker discovery study [37], were used to corroborate already discovered DMD biomarkers. In addition, orthogonal protein quantification methods were used to quantify two DMD biomarkers.

The majority of the serum DMD biomarkers discovered within the past decade have been identified using affinity-based discovery [29, 31], urging for validation using non-affinity-based technologies operating within the same range of sensitivity. Introducing orthogonal methods in the validation step enables the identification of false positive biomarker candidates detected due to unspecific interactions by the affinity reagents. Using PRM-MS, five out of ten biomarker candidates were confirmed (Fig. 2 and Supplementary Fig. 2). Four of these, CA3 [29, 34, 37, 38, 60], LDHB [37, 38], MYL3 [29, 37, 38] and FGG [51], exhibit an association with DMD and a strong age dependency, in accordance with previous reports [29, 37, 60]. In contrast, TNNT3 [29, 38], ETFA [29, 38], MDH2 [29, 37, 38], NES [38] and MAP4 [38] previously identified as biomarkers in both plasma and serum and consistent across 4 cohorts [29, 37], were not detected. One possible explanation could lie in the inherent differences in the two detection methods. The concentrations of serum CA3 and MYL3 in the ng/ml range, are in accordance with concentrations previously published in DMD and control studies [61–63], further corroborating our findings.

Quantification of proteins faces great challenges due to inherent characteristics such as the existence of posttranslational modifications, splice-isoforms and degradation products, all of which may interfere with their detection. While both methods rely on the detection of rather short protein fragments, peptides generated through enzymatic digestion in the case of MS and epitopes recognized by antibodies in the case of antibody-based methods, the measured signals are not necessarily based on the detection of identical fragments. While the detection of proteins by antibody-based technologies is dependent on whether the short amino-acid sequence comprising the epitope is accessible for the antibody used, PRM-MS requires the targeted peptide to be intact, unmodified and sufficiently ionized for detection [64]. In addition to the concentration and availability of epitopes in samples, immuno-based methods are also limited by the specificity of the antibodies used. Although the antibodies used in previous studies have been validated using at least three proteomics methods, their performance and ability to discriminate between the true target and interfering molecules is context dependent [41]. Using this orthogonal strategy, the identification and quantification of protein biomarkers can be achieved unbiased by the detection mechanism that the technological platforms rely on.

Proteins related to muscle function and muscle tissue necrosis are repeatedly reported in DMD biomarker discovery studies [29, 31, 65, 66], where they are found to be upregulated in young, ambulant DMD patients compared to controls. The biomarkers confirmed in this study are highly relevant in the context of DMD and together could mirror pathological changes. CA3 and LDHB, along with CK and troponins such as TNNT3, have repeatedly been described as serum markers of muscle damage during extensive exercise [67]. In addition, as the synthesis of CA3 and CK-M in skeletal muscle is constant over time in DMD patients, these biomarkers could reflect the muscle mass or the membrane integrity of muscle cells [68]. MYL3 is predominantly expressed in cardiac cells, and elevated serum levels of MYL3 have been reported as a biomarker of cardiomyocyte necrosis [69]. Continuous muscle damage in muscle dystrophies has been observed to cause inflammation and fibrinogen deposition around the damaged tissue, leading to upregulation of collagen and onset of fibrosis [70]. The gamma subunit of fibrinogen, FGG, might be used here to provide insight in fibrosis formation. The continuous muscle degeneration and the progression of fibrosis experienced by the patients makes age a confounding factor. Our results corroborate previous findings that CA3, LDHB, MYL3 and FGG are associated with DMD and can discriminate between ambulant and non-ambulant patients and controls regardless of whether age is accounted for. However, only LDHB is able to discriminate between ambulant and non-ambulant patient groups when age is accounted for. LDHB has previously been shown to be associated with respiratory capacity and forced vital capacity [38].

Expression of CA3, MYL3, LDHB and FGG, on both transcript and protein levels (Human Protein Atlas project, www.proteinatlas.org) [45], reveals that all proteins except for FGG are highly expressed in skeletal and heart muscle. Expression of MYL3 and CA3 is rather restricted to muscle tissue and could indicate a muscle origin. CA3 is enriched in slow-twitching type I fibers and as a metabolic enzyme involved in maintenance of intracellular pH homeostasis [71]. MYL3 is involved in muscle function and MYL3 gene mutations are associated with Hypertrophic cardiomyopathy [72]. Furthermore, MYL3 has been shown to be exported by a lysosomal-associated membrane protein, LAMP1, from *mdx* myotubes that may explain its presence in the blood stream [73]. In contrast to CA3 and MYL3, LDHB is an enzyme involved in anaerobic metabolism and is more ubiquitously expressed on an anatomic level [45]. FGG is involved in hemostasis and thrombosis, mainly expressed in liver and subsequently secreted [74]. There is evidence of fibrinogen disposition around dystrophic muscle tissue in DMD which may be driving inflammation and fibrosis formation [75]. In addition, serum FGG has been reported to predict cardiovascular disease [76]. The expression and function of these biomarkers may reflect alterations in different tissues and biological processes. If further validated, these biomarkers together would reflect not only muscle injury but also inflammation, metabolic changes in energy production as well as predict cardiac involvement. As shown previously [38] and in this study, the abundance trajectories of the biomarkers, like MYL3, over time will reach the serum levels of healthy individuals, although progression of the disease still persist. Using only one biomarker to monitor disease progression in DMD might not be specific and sensitive enough to capture the disease progression over time.

In this study, SIS-PrESTs developed within the Human Protein Atlas project were used for quantification of biomarkers. Many of these protein fragments have also been used as antigens for the large-scale production of monospecific antibodies [39, 45] generating a valuable resource of paired proteins and corresponding antibodies. These reagents constitute valuable tools as their use in orthogonal proteomics methods reduces the complexity of the analysis, narrowing down the detected units and increasing the comparability of the results. While MS dominates the discovery field and has been hypothesized to become readily used in the clinic in the future [77], immunoassays still dominate clinical tests [4]. Therefore, we suggest the strategy outlined for analytical validation of additional biomarkers within DMD.

## Electronic supplementary material

Below is the link to the electronic supplementary material.

Additional file 1: Supplementary Fig. 1. Total protein concentrations in serum samples; Supplementary Fig. 2. Scatter plots representing biomarker abundance measured using the suspension bead array platform. Supplementary Fig. 3. Standard curves and carbonic anhydrase 3 quantification of samples in six replicates using sandwich immunoassay.

## Data Availability

All data can be accessed through https://panoramaweb.org/orthogonal_dmd.url

## Abbreviations

AGC: Automatic Gain Control
CA3: Carbonic anhydrase 3 (CA3)
CK: Creatine kinase
CK-M: Creatine kinase muscle isoform
COL1A1: Collagen alpha-1(I) chain
DMD: Duchenne Muscular Dystrophy
DMSO: Dimethylsulfoxide
ETFA: Electron transfer flavo-protein A
FGG: Fibrinogen gamma chain
LDHB: Lactate dehydrogenase B
LUMC: Leiden University Medical Center
MDH2: Malate dehydrogenase 2
MAP4: Microtubule-associated protein 4
MS: Mass spectrometry
MYL3: Myosin light chain 3
NCE: Normalized Collision Energy
NES: Nestin
QTag: N-terminal quantification tag
PBS: Phosphate-buffered saline
PBST: Phosphate-buffered saline/Tween
PrEST: Protein epitope signature tag
PRM-MS: Parallel Reaction Monitoring Mass Spectrometry
PQN: Probabilistic quotient normalization
SIS-PrEST: ^13^C^15^N-isotope labeled standards
TNNT3: Troponin type T 3
