# Supplementary material for: Orthogonal proteomics methods warrant the development of Duchenne muscular dystrophy biomarkers
Source: Clin Proteomics. 2023 Jun 12;20:23. doi: 10.1186/s12014-023-09412-1 (PMC10258980; doi:10.1186/s12014-023-09412-1)
Supplement: Supplementary file 1 — Additional file 1: Supplementary Fig. 1. Total protein concentrations in serum samples; Supplementary Fig. 2. Scatter plots representing biomarker abundance measured using the suspension bead array platform. Supplementary Fig. 3. Standard curves and carbonic anhydrase 3 quantification of samples in six replicates using sandwich immunoassay. [file 12014_2023_9412_MOESM1_ESM.pdf]

Supplementary figures and tables

**Supplementary figure 1.** Total protein concentration in serum samples. (A) Boxplot displaying total protein concentration estimated by BCA assay in healthy controls (open circles) and DMD patients (closed circles). (B) Scatter plot representing distribution of total protein concentration across age in serum from DMD patients and healthy donors in the same age range.

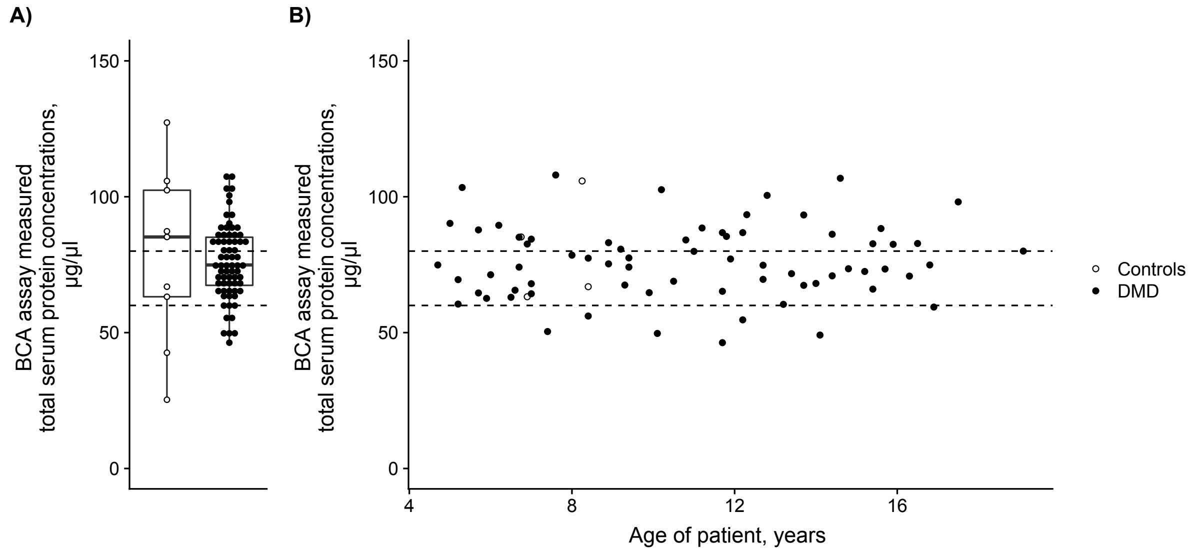

**Supplementary figure 2.** Scatter plots representing biomarker abundance measured using the suspension bead array platform. Abundance is measured as Median Fluorescent Intensities for MYL3 (A), CA3 (B), LDHB (C) and COL1A1 (D) .

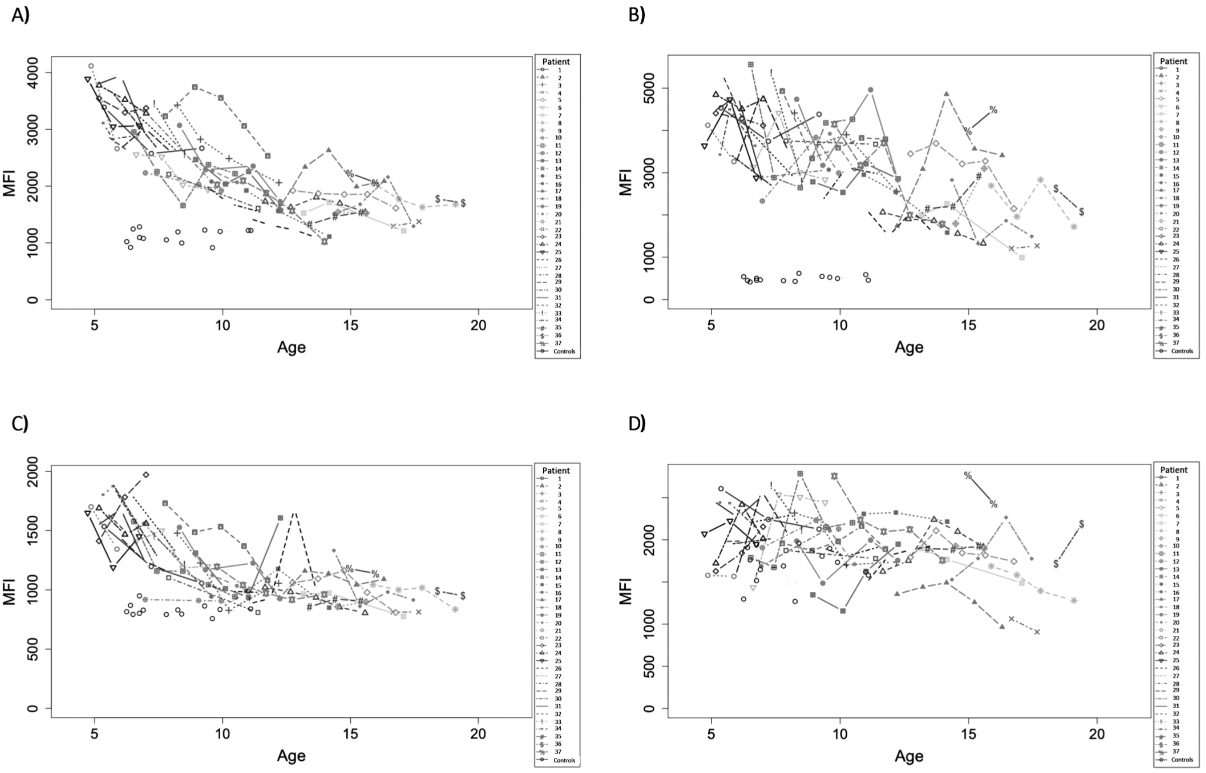

**Supplementary figure 3.** Standard curves and carbonic anhydrase 3 quantification of samples in six replicates (A-F) using sandwich immunoassay. Standard diluents are denoted with open circles, four-parametric log-logistic regression curve (4PL) model shown with a line, and calculated CA3 concentrations for each quantifiable sample denoted with closed circles.

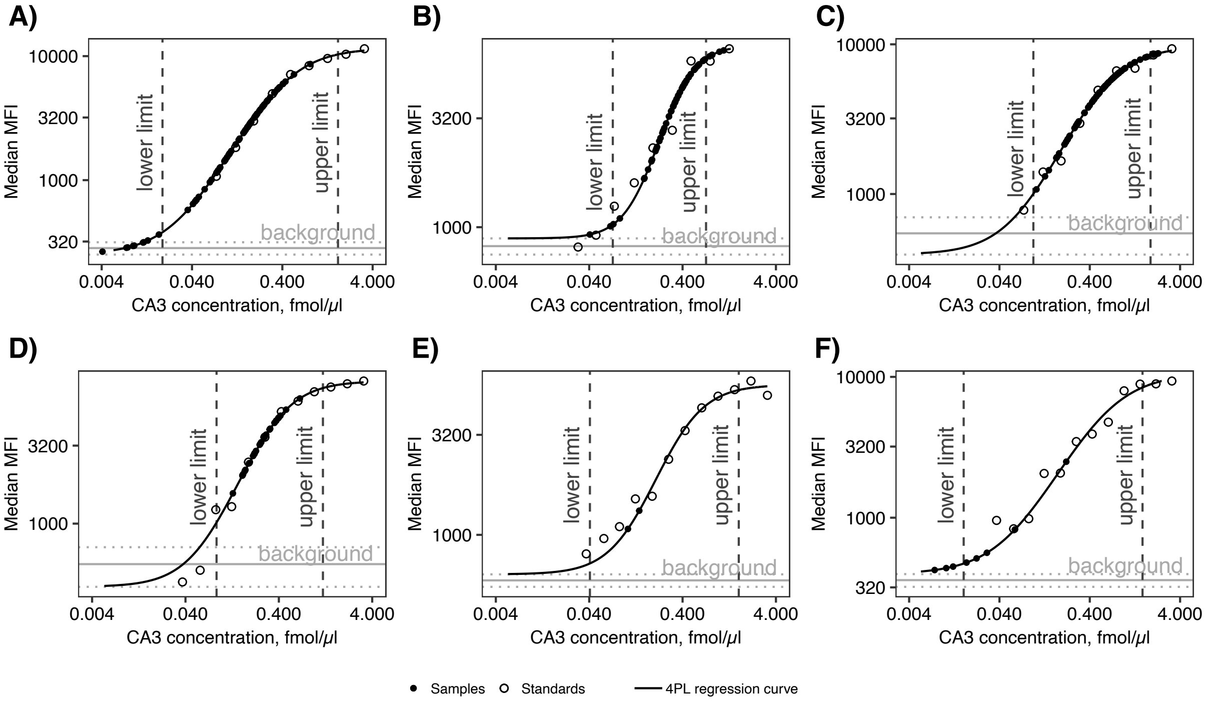


| Curve | No. Quantified Samples | Samples | Sample Dilution | Highest CA3 standard diluent, fmol/µl | Lowest CA3 standard diluent, fmol/µl | Number of standard diluents | Background MFI | Bead stock batch | Bead stock dilution | Biotinylated antibody batch | Biotinylated antibody dilution | Diluted SAPE batch | Dilution guinea-pig serum | Batch standard diluents |
|---|---|---|---|---|---|---|---|---|---|---|---|---|---|---|
| A) | 71 | DMD+CONT | ~500x | 3,2 | 0,068 | 9 | 283 ± 32 | A | 8x | A | 150x | A | 200x | A |
| B) | 53 | DMD+CONT | ~200x | 1,3 | 0,003 | 9 | 817 ± 70 | B | 7x | B | 120x | B | 200x | A |
| C) | 71 | DMD | ~200x | 3,2 | 0,068 | 9 | 546 ± 152 | B | 12x | B | 120x | B | 200x | A |
| D) | 29 | DMD with CA3 > 130 fmol/µl | ~500x | 3,2 | 0,003 | 12 | 547 ± 157 | C | 7x | C | 150x | C | 500x | B |
| E) | 2 | CONT | ~100x | 3,2 | 0,003 | 12 | 589 ±43 | C | 7x | C | 150x | C | 100x | C |
| F) | 5 | CONT | ~50x | 3,2 | 0,003 | 12 | 360 ± 37 | D | 8x | D | 150x | D | 50x | D |
